# Supplementary material for: Active site specificity profiling datasets of matrix metalloproteinases (MMPs) 1, 2, 3, 7, 8, 9, 12, 13 and 14
Source: Data Brief. 2016 Feb 22;7:299–310. doi: 10.1016/j.dib.2016.02.036 (PMC4777984; doi:10.1016/j.dib.2016.02.036)
Supplement: Supplementary file 10 — Supplementary material [file mmc10.zip › WebPICS_hMMP8_G_1%/P1prime.html]

 

PICS results


|  |  |
| --- | --- |
| **P1prime\_C**  7 in 120 sites   5.8 %    effects > 10 perc. pnts.  (vice-versa in brackets)  P2\_L: 29.6 (13.0)   P2\_Y: 21.9 (19.2)   P3prime\_V: 20.3 (14.2) |  |
| **P1prime\_F**  7 in 120 sites   5.8 %    effects > 10 perc. pnts.  (vice-versa in brackets)  P3\_H: 38.7 (54.2)   P2\_F: 21.1 (16.4)   P2\_W: 39.6 (69.2)   P1\_N: 37.9 (11.6)   P1\_P: 35.4 (27.5)   P2prime\_Q: 32.9 (19.2)   P3prime\_V: 34.6 (24.2) |  |
| **P1prime\_L**  49 in 120 sites   40.8 %    effects > 10 perc. pnts.  (vice-versa in brackets)  P3\_P: -21.3 (-37.2)   P2prime\_K: 11.9 (23.2) |  |
| **P1prime\_Q**  12 in 120 sites   10.0 %    effects > 10 perc. pnts.  (vice-versa in brackets)  P3\_P: 43.4 (18.6)   P2\_A: 21.6 (18.6)   P1\_A: 22.5 (20.8) |  |
| **P1prime\_V**  12 in 120 sites   10.0 %    effects > 10 perc. pnts.  (vice-versa in brackets)  P3\_P: 35.0 (15.0)   P2\_F: 17.5 (23.3)   P2\_M: 13.4 (40.0)   P1\_Q: 15.8 (17.3)   P2prime\_I: 14.2 (13.1)   P3prime\_N: 16.7 (20.0) |  |
| **P1prime\_Y**  4 in 120 sites   3.3 %    effects > 10 perc. pnts.  (vice-versa in brackets)  P2\_A: 38.3 (11.0)   P2\_H: 22.5 (30.0) |  |
